# The Important Role of FLT3-L in Ex Vivo Expansion of Hematopoietic Stem Cells following Co-Culture with Mesenchymal Stem Cells

**DOI:** 10.22074/cellj.2016.3715

**Published:** 2015-07-11

**Authors:** Farhad Oubari, Naser Amirizade, Hemn Mohammadpour, Mozhdeh Nakhlestani, Mahin Nikougoftar Zarif

**Affiliations:** 1 Blood Transfusion Research Center, High Institute for Research and Education in Transfusion Medicine, Tehran, Iran; 2 Faulty of Paramedics, Kermanshah University of Medical Science, Kermanshah, Iran; 3 Department of Medical Immunology, Faculty of Medical Science, Tarbiat Modares University, Tehran, Iran

**Keywords:** Fms-Related Tyrosine Kinase 3 Ligand, Hematopoietic Stem Cells, Mesenchymal Stem Cells, Expansion

## Abstract

**Objective:**

Hematopoietic stem cells (HSCs) transplantation using umbilical cord blood (UCB) has improved during the last decade. Because of cell limitations, several studies focused on the *ex vivo* expansion of HSCs. Numerous investigations were performed to introduce the best cytokine cocktails for HSC expansion The majority used the Fms-related tyrosine kinase 3 ligand (FLT3-L) as a critical component. According to FLT3-L biology, in this study we have investigated the hypothesis that FLT3-L only effectively induces HSCs expansion in the presence of a mesenchymal stem cell (MSC) feeder.

**Materials and Methods:**

In this experimental study, HSCs and MSCs were isolated from UCB and placenta, respectively. HSCs were cultured in different culture conditions in the presence and absence of MSC feeder and cytokines. After ten days of culture, total nucleated cell count (TNC), cluster of differentiation 34+(CD34^+^) cell count, colony forming unit assay (CFU), long-term culture initiating cell (LTC-IC), homeobox protein B4 (*HoxB4*) mRNA and surface CD49d expression were evaluated. The fold increase for some culture conditions was compared by the t test.

**Results:**

HSCs expanded in the presence of cytokines and MSCs feeder. The rate of expansion in the co-culture condition was two-fold more than culture with cytokines (P<0.05). FLT3-L could expand HSCs in the co-culture condition at a level of 20-fold equal to the presence of stem cell factor (SCF), thrombopoietin (TPO) and FLT3-L without feeder cells. The number of extracted colonies from LTC-IC and CD49d expression compared with a cytokine cocktail condition meaningfully increased (P<0.05).

**Conclusion:**

FLT3-L co-culture with MSCs can induce high yield expansion of HSCs and be a substitute for the universal cocktail of SCF, TPO and FLT3-L in feeder-free culture.

## Introduction

Hematopoietic stem cells (HSCs) are adult stem cells that have the capacity for self-renewal, proliferation and differentiation into hematopoietic cell lineages (1). The tremendous ability of HSCs in hemostasis regulation is due to numerous molecules and soluble factors that affect HSC behavior (2). Adhesion molecules, growth factors and cell-cell interactions in a specific niche are crucial factors that balance expansion, differentiation and migration of HSCs (3). Attempts for *ex vivo* expansion of HSCs in order to improve clinical outcomes of HSCs transplantation, especially on cord blood units has been considered in the last decade (4). One of the concerns about HSCs *ex vivo* expansion with growth factors is the production of short term reconstituting and nondurable HSCs that affect transplantation outcome (5). Based on previous studies of several recognized ligands and respective receptors, receptor-type tyrosine kinas (RTK) class III and its ligands have dominant roles in hematopoiesis and HSCs expansion (6). Fmsrelated tyrosine kinase 3 ligand (FLT3-L) is one of the RTKs produced in the bone marrow, thymus and liver; its binding to FLT3 improves HSCs expansion (7). Numerous investigations have been performed to introduce the best cytokine cocktails for HSCs expansion. In the majority, FLT3-L was used as a critical component (8, 9). FLT3-L causes over expression of very late antigen 4 (VLA4) and VLA5 on the HSCs surface and consequently more adhesion of HSCs to mesenchymal stem cells (MSCs) and cells which express vascular cell adhesion molecule-1(VCAM-1) and intracellular adhesion molecule-1 (ICAM-1) (7). One of the primary, important cells in bone marrow niches are MSCs (10). MSCs support HSCs maintenance and expansion through secretion of growth factors, adhesion and signal transduction (11, 12). According to FLT3-L biology, in the present study we have investigated the effect of FLT3-L on HSCs expansion co-cultured with MSCs as a feeder layer compared to enriched culture medium. In addition, increased expression of homeobox protein B4 (*HoxB4*) as a transcription factor in HSCs expansion has been reported (13), thus we also assessed the level of *HoxB4* in different culture conditions with and without FLT3-L.

## Materials and Methods

### Isolation of cluster of differentiation 34^+^ (CD34^+^) hematopoietic stem cells

In this experimental study, venous UCB was collected from three healthy donors, full term neonates in collection bags (JMS, Korea) that contained 22 ml anti coagulation reagent. All the donors signed informed consent. Briefly, low density UCB mononuclear cells were isolated by Ficoll Hypaque (density: 1077 g/cm^3^, Pharmacia, Sweden) under density gradient centrifugation. CD34^+^ cells were enriched from mononuclear cells using bead conjugated anti-CD34 antibody (Miltenyi Biotec, Germany) with the Magnetic Activated Cell Sorting (MACS) method according to the manufacturer’s instructions (Miltenyi Biotec, Germany). The efficiency of purification was verified by flow cytometry (Partec PAS III, Germany) of counterstained sorted cells with phycoerythrin (PE) conjugated anti-CD34 (Dako, Denmark) and fluorescein isothiocyanate (FITC) conjugated CD38 (Dako, Denmark). Non-specific reactions were excluded using isotype controls. The samples that contained HSCs with low expression of CD38 (<15% positive) were selected.

### Isolation of mesenchymal stem cells from placenta

Placenta tissue was obtained from healthy donor mothers following informed consent. After complete drainage of cord blood, we excluded the deciduae and carefully dissected the remaining placental tissue under sterile conditions. The collected pieces were twice washed with phosphatebuffered saline (PBS, Sigma, USA), mechanically minced and enzymatically digested in 0.1% collagenase for 2 hours (Sigma, USA). To remove undigested fragments, the cell suspension was filtered through a membrane that had a 70 μm pore size. Red cells were lysed using lysing reagent (BD Pharmingen, USA). Homogenized cells were subsequently washed and cultured in T75 Dulbecco’s modified eagle medium (DMEM, Sigma, USA) with 1% glucose supplemented by 10% fetal bovine serum (FBS, Sigma, USA). The media was changed each three days and cells were passage until they were 80% confluent. Passage-3 cells were characterized using FITC conjugated CD45, CD90, CD29, CD271, CD44 and PE conjugated CD34, CD73, CD105 and CD166 monoclonal antibodies (Dako, Denmark or BD Pharmingen, USA). Also the differential capacity of isolated cells toward osteocytes and adipocytes was performed using the recommended culture medium (Sigma, USA) after which differentiation was evaluated via oil red-O and alizarin red staining (Sigma, USA), respectively.

### Cytokines

Recombinant FLT3-L, thrombopoietin (TPO) and stem cell factor (SCF) were purchased from Stem Cell Technologies (Canada) and used at a concentration of 100 ng/ml.

### Culture of hematopoietic stem cells in different conditions

CD34^+^ cells were cultured in Stemspan™ serum-free expansion medium (Stem Cell Technologies, Canada). The cytokines were used as follows: one group received FLT3-L, TPO and SCF; another group received TPO and SCF; and the third group only received FLT3-L (all at a concentration of 100 ng/ml). HSCs were additionally cultured in Stemspan™ serum-free medium without any cytokines as the control group. These conditions were repeated in a co-culture condition with the placental derived MSCs. An irradiated (16 G/30 minutes) MSCs layer at 90% confluency was used as feeder cells. After 10 days of culture, total nucleated cell count (TNC) and CD34^+^/CD38- cells were counted and the fold increase under different culture conditions was evaluated.

### Relative real-time polymerase chain reaction (PCR)

HSCs were isolated from MSCs by the MACS method in the day of 1 and 10 after culture. Total RNAs were extracted using TRIzol reagent (Qiagen, Germany) and cDNA synthesized from the obtained RNA (Bioneer, USA). Relative real-time PCR was performed for *HoxB4* using matched primers with the following sequences:

forward: TCCCACTCCGCGTGCAAAGA and reverse: AAGACCTGCTGGCGCGTGTA.

*GAPDH* mRNA was measured as the housekeeping control.

### Hematopoietic colony formation

The colony forming unit assay (CFU) was performed using Methocult medium. Briefly, 2×10^3^ CD34^+^ cells were cultured in Methocult medium that contained supplemented cytokines according to the manufacturer’s instructions for 14 days at 37˚C and 5% CO_2_ (Stem cell Technologies, Canada). CFU were calculated using an invert microscope to visualize clusters that consisted of 40 or more cells.

### Human long-term culture-initiating cell (LTC-IC) assay

LTC-IC assay was performed to determine the presence of long-term HSCs. For this purpose, 5×10^3^ CD34^+^ cells at the day of isolation and after 10 days of treatment were cultured in Myelocult M5300 (Stem Cell Technologies, Canada) on an irradiated stromal cell feeder and incubated at 37˚C and 5% CO_2_. The medium was changed every four days. After six weeks of culture, the cells were suspended with trypsin and 2×10^3^ cells were cultured in Methocult for the CFU assay. Colonies were counted after 14 days of culture as long-term HSCs by invert microscope.

### CD49d flow cytometry assay

CD49d flow cytometry assays were conducted using FITC conjugated anti-CD49d and a mouse isotype control. Briefly, cells were washed with PBS and re-suspended at a concentration of 106 cells/ml. After 30 minutes incubation at room temperature, cells were immediately analyzed. The dead cells were excluded with propidium iodide (Sigma, USA).

### Statistical analysis

All experiments were done in triplicate. The results of this experimental study are represented as mean ± standard error measurement (SEM). Data analysis was performed by the paired Student’s t test and analysis of variance using SPSS (IBM,USA) 16. P<0.05 was considered statistically significant.

## Results

### Purity of CD34^+^ isolated cells

**Fig.1:**
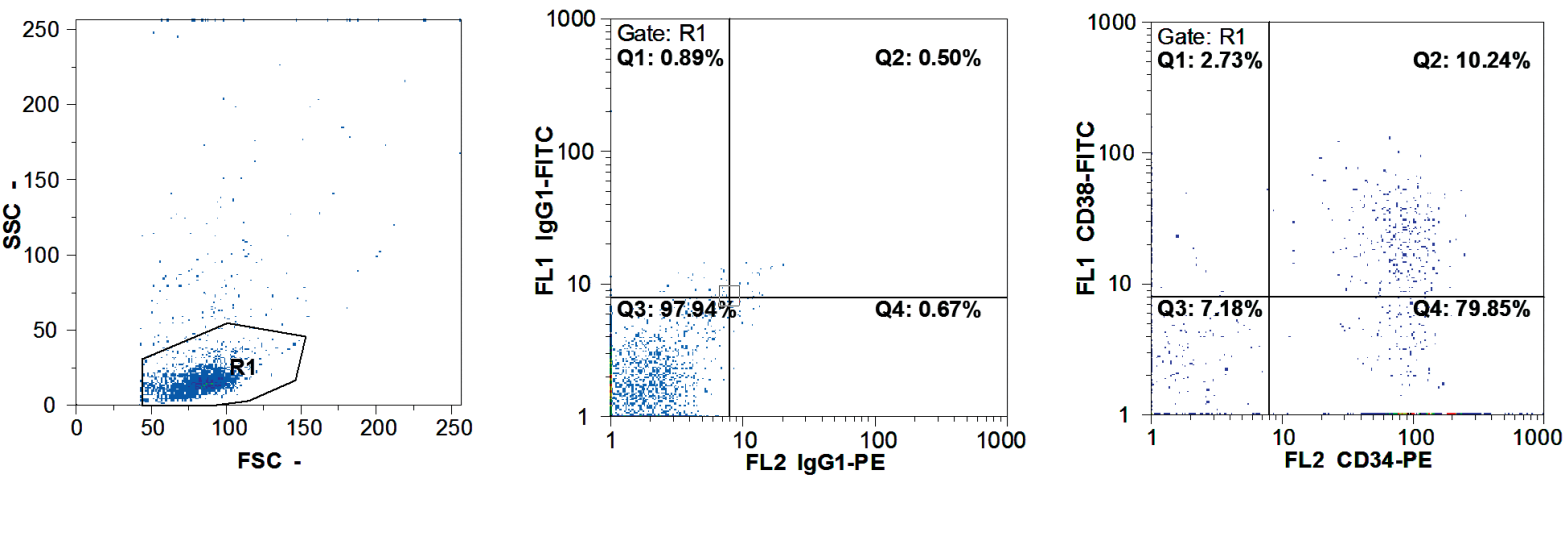
Purity of isolated CD34^+^ cells. From the left: cell scattering, isotype control and CD34-phycoerythrin (PE) versus CD38-fluorescein isothiocyanate (FITC) populations. SSC; Side scatter, FCS; Forward scatter, CD; Cluster of differentiation and FL; Fluorescent.

Flow cytometric evaluation of isolated cells confirmed 89.6 ± 8.8% purity for the CD34^+^/CD38- population. Less than 12.3% of cells expressed dim CD38 surface marker (Fig.1).

### Mesenchymal stem cells characterization

**Fig.2:**
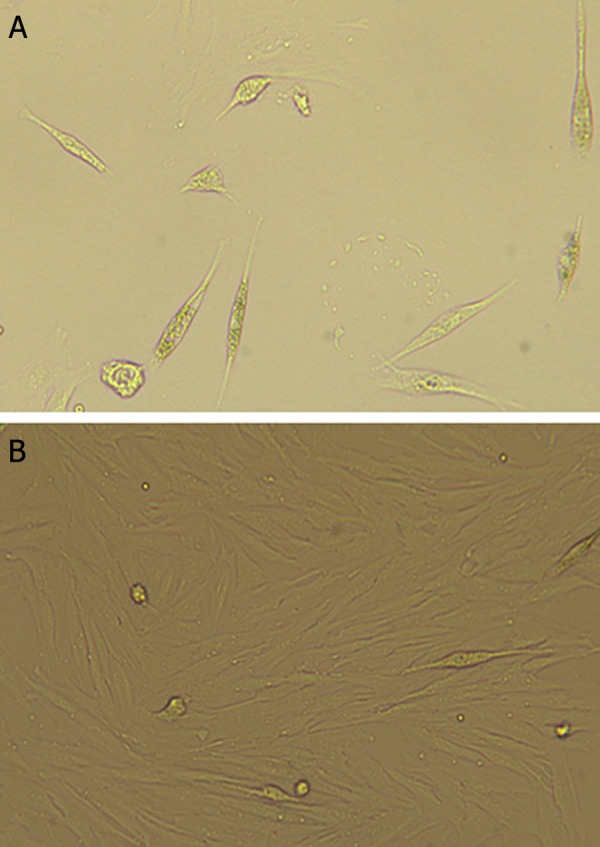
Morphology of mesenchymal stem cells. A. Two days after isolation and B. 28 days after isolation in passage-3 (magnification: ×200).

**Fig.3:**
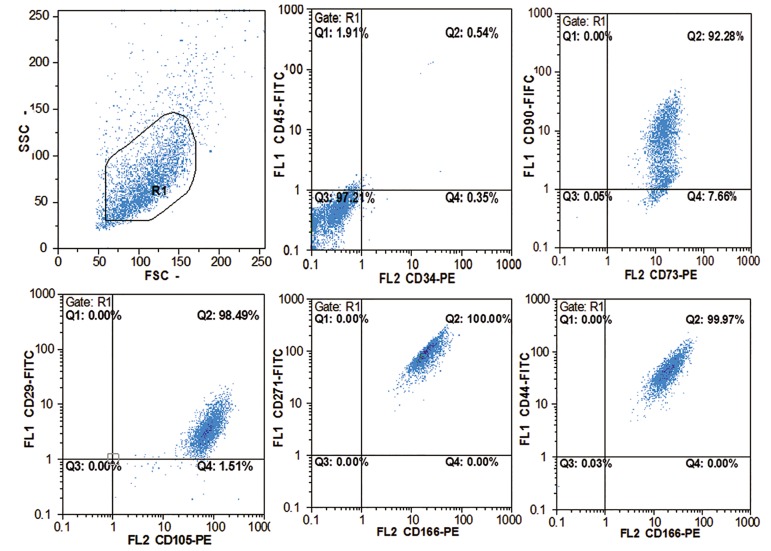
Immunophenotyping of mesenchymal stem cells. More than 95% of cells were gated and regions were adjusted based on the isotype control reactivity. SSC; Side scatter, FCS; Forward scatter, FL; Fluorescent, FITC; Fluorescein isothiocyanate, PE; Phycoerythrin and CD; Cluster of differentiation.

After morphological assessments (Fig.2), MSCs from passage 3 were selected for immunophenotyping. MSCs were positive for CD90, CD271, CD44, CD73, CD29, CD166 and CD105. In addition they were negative for CD34 and CD45 which confirmed their phenotype (Fig.3).

**Fig.4:**
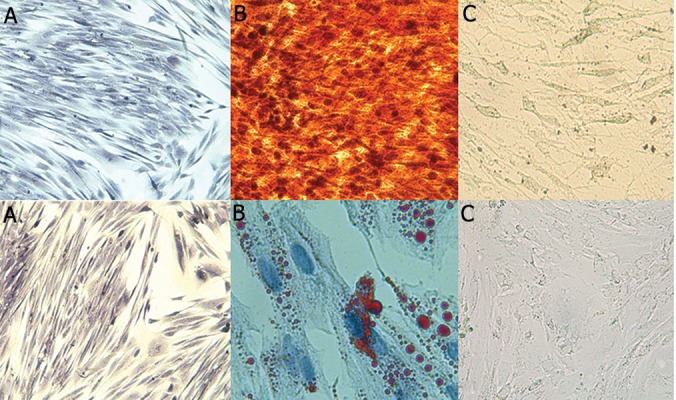
Specific staining of differentiated mesenchymal stem cells. Upper row: osteogenic differentiation. Lower row: adipogenic differentiation. A. Undifferentiated cells with specific staining (×100), B. Differentiated cells with specific staining and C. Differentiated cells without staining (×200).

Microscopic examination of MSCs morphology confirmed osteogenic and adipogenic changes in the cells. Furthermore, exact evaluation of differentiation capacity was confirmed by the positive reactions of alizarian red in osteogenic and oil red-O in adipogenic differentiated cells (Fig.4).

### Total nucleated cell count and CD34^+^ cell counts in different culture conditions

At 10 days after HSCs culture with and without MSCs in combination with different cytokine supplements, we performed a TNC on the CD34^+^/ CD38- cell populations. In all co-cultures TNC increased compared to feeder-free culture conditions (P<005, Table 1).

FLT3-L with MSCs feeder clearly increased TNC count compared to the respective group without feeder (P=0.002). Co-culture of cells with three cytokines showed a 74-fold TNC increase whereas the culture with only FLT3-L showed a 24-fold TNC increase (P<0.05). This criteria in the presence of SCF and TPO showed only a 47- fold increase in co-culture compared to the feederfree condition which had a 38-fold increase.

**Fig.5:**
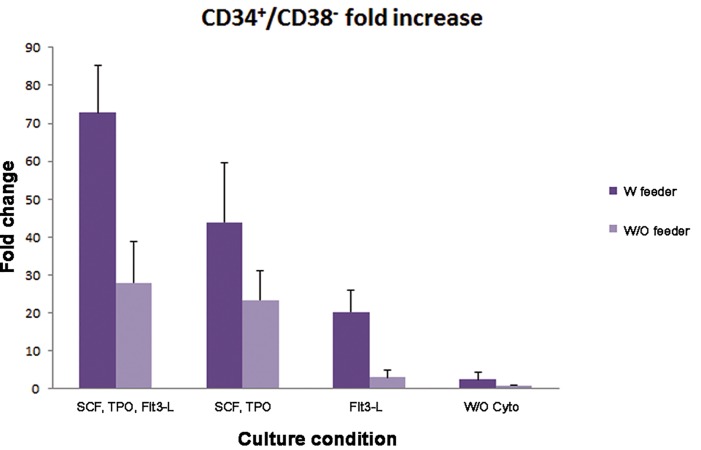
CD34^+^/CD38- fold increase in different culture conditions. SCF; Stem cell factor, TPO; Thrombopoietin, Flt3-L; Fms-related tyrosine kinase 3 ligand, W/O cyto; Without cytokine and CD; Cluster of differentiation.

The CD34^+^/CD38- cell count confirmed more expansion of these cells in the co-culture condition (P<0.05). Of note, the effect of FTL3-L on CD34^+^/ CD38- showed a 20.18-fold increase in co-culture with MSCs, which was as high as three cytokines in feeder-free culture (27.09) but not significant (P>0.06, Fig.5).

**Table 1:** TNC and CD34+/CD38- cell count and fold increase after ten days of culture in different groups


Culture conditions	Without feeder				With feeder			
	SCF, TPO,FLT3-L	SCF, TPO	FLT3-L	W/O cyto	SCF, TPO,FLT3-L	SCF, TPO	SCF, TPO	SCF, TPO

TNC×10^6^	4.35 ± 0.92	3.82 ± 0.15	0.29 ± 0.12	0.06 ± 0.03	7.37 ± 1.55	4.74 ± 1.01	2.43 ± 0.60	0.31 ± 0.10
TNC fold increase	43 ± 9	38 ± 15	3 ± 1	0.6 ± 0.03	74 ± 17	47 ± 10	24 ± 6	3 ± 1
CD34^+^/CD38^-^×10^6^	2.3 ± 0.84	1.76 ± 0.39	0.08 ± 0.002	0.02 ± 0.004	6.01 ± 1.15	3.63 ± 0.99	1.67 ± 0.42	0.19 ± 0.07
CD34^+^/CD38^-^fold increase	28 ± 9	23 ± 4	3 ± 1	0.9 ± 0.04	73 ± 11	44 ± 10	20 ± 5	2 ± 0.7


TNC; Total nucleated cell count, SCF; Stem cell factor, TPO; Thrombopoietin, FLT3-L; Fms-related tyrosine kinase 3-Ligand, W/O cyto; Without cytokine and CD; Cluster of differentiation.

### Colony forming unit assay

**Fig.6:**
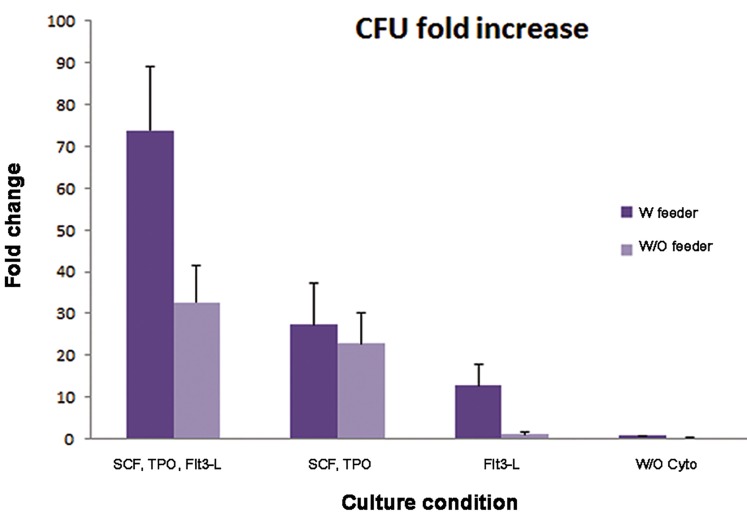
Colony forming unit fold increase in different culture conditions. SCF; Stem cell factor, TPO; Thrombopoietin, Flt3-L; Fms-related tyrosine kinase 3 ligand and W/O cyto; Without cytokine.

The CFU assay was performed using Methocult media. We observed more colonogenic capacity in cells with feeder in co-culture conditions compared to the same conditions without feeder (P<0.05), except when TPO and SCF were added as growth factors and without cytokine. Like the TNC and CD34^+^/CD38- cells fold increase, The CFU assay showed meaningful increase in co-culture of HSCs with MSC in the presence of FLT3-L condition, than the corresponding group (P<0.05, Fig.6).

### Long-term culture-initiating cell assay

**Fig.7:**
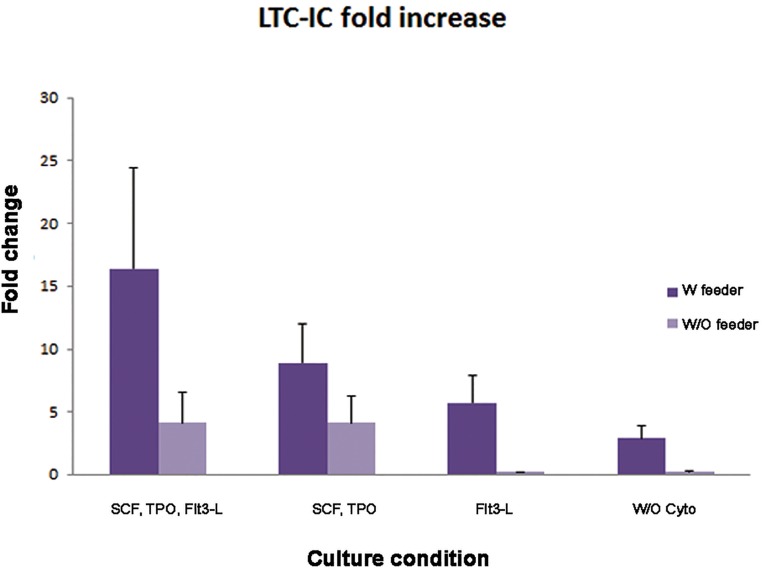
Long-term culture initiating cells (LTC-IC) fold increase in different culture conditions. SCF; Stem cell factor, TPO; Thrombopoietin, Flt3-L; Fms-related tyrosine kinase 3 ligand and W/O cyto; Without cytokine.

The LTC-IC assay was performed for six weeks to measure primitive HSCs based on their capacity to produce myeloid progenitors in different culture conditions, after which colony formation of cells confirmed the presence of long-term culture cells. Of the fold increase in groups that had different combinations of the mentioned cytokines, only LTC-IC in the group with MSC feeder alone showed significant improvement (P<0.05, Fig.7). The combination of FLT3-L with MSCs caused more improvement in LTC-IC compared to the cocktail of FLT3-L, TPO and SCF without feeder (5.1 vs. 4.7).

### CD49d expression

**Fig.8:**
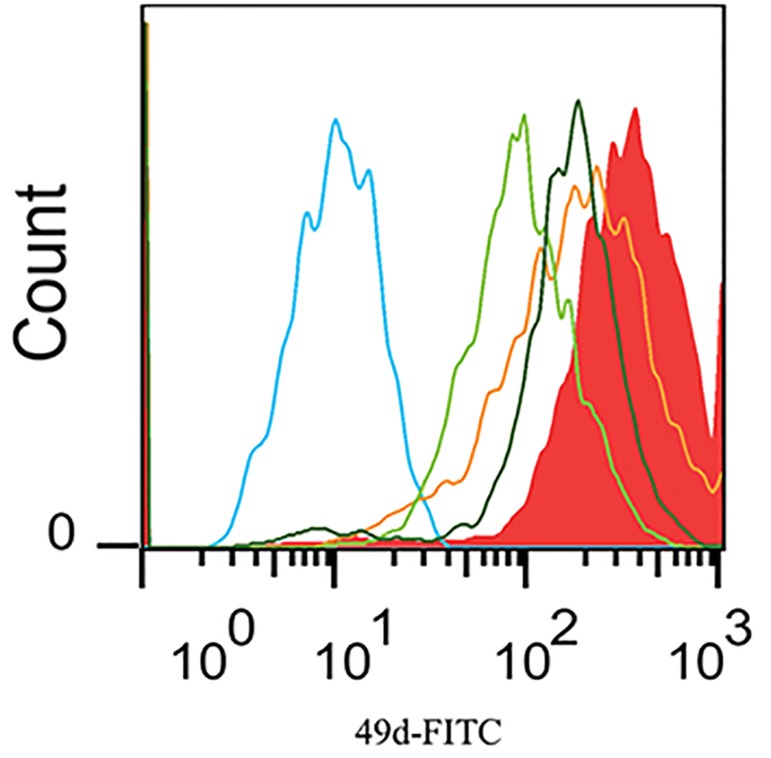
Flow cytometry overlay histogram of CD49d adhesion molecule in expanded hematopoietic stem cells in different culture conditions. Coculture of HSCs with mesenchymal stem cells plus Fms-related tyrosine kinase 3- ligand induced CD49d expression more than the other groups. Left to right: culture of HSCs by thrombopoietin, stem cell factor and FLT3-L. Culture of HSCs with TPO and SCF. Pre-treatment group. Co-culture of HSCs with MSCs. Co-culture of HSCs with MSCs and FLT3-L. FITC; Fluorescein isothiocyanate and CD; Cluster of differentiation.

We investigated the effects of MSCs plus FLT3-L and cytokine cocktails on CD49d adhesion molecule expression in HSCs (Fig.8). Expression of CD49d in FLT3-L combined with MSCs was significantly greater than the other groups (P<0.001). Expression of CD49d in the groups that only received cytokine cocktail remarkably decreased compared to the pre-treatment group (P<0.001). Our results showed that expansion of HSCs with cytokines negatively changed CD49d expression. The group treated with three cytokines (TPO, SCF and FLT3-L) had the lowest expression of CD49d which was noticeably less than the other groups (P<0.001).

### HoxB4 gene expression

*HoxB4* protein is one of the major regulators of HSCs proliferation that increases proliferative capacity of HSCs without any lineage differentiation. Real-time PCR analysis of *HoxB4* gene expression in contrast with *GAPDH* showed high mRNA in the groups with cytokines combined with MSCs compared to the control groups (Table 2). Cytokine cocktails of FLT-3 L, TPO and SCF with MSCs as feeder led to significantly increased *HoxB4* expression in HSCs (P<0.05).

**Table 2:** Homeobox B4 (HOXB4) gene expression in different groups in contrast to GAPDH


Culture conditions	Without feeder				With feeder			
	W/o cyto	FLT3-L	SCF, TPO	SCF, TPO,FLT3-L,	W/o cyto	FLT3-L	SCF, TPO	SCF, TPO,FLT3-L,

Culture conditions	4.35 ± 0.92	3.82 ± 0.15	0.29 ± 0.12	0.06 ± 0.03	7.37 ± 1.55	4.74 ± 1.01	2.43 ± 0.60	0.31 ± 0.10
HoxB4 fold expression	28 ± 9	23 ± 4	3 ± 1	0.9 ± 0.04	73 ± 11	44 ± 10	20 ± 5	2 ± 0.7


SCF; Stem cell factor, TPO; Thrombopoietin; FLT3-L; Fms-related tyrosine kinase 3-Ligand and W/0 cyto; Without cytokine.

## Discussion

Although cord blood transplantation holds prominent advantages, limited stem cells affect successful transplantation (14). In the recent decade several studies have researched diverse approaches and protocols to improve HSCs expansion (15). Although remarkable expansion of highly purified CD34^+^ cells has been previously described with different cocktails of cytokines, LTC-IC data showed mild to moderate repopulation of stem cells (16). It has been shown that expansion of HSCs with cytokines changes the expression of adhesion and homing molecules and may restrict the homing and engraftment capabilities of HSCs (17).

The crucial role of adhesion molecule of CD49d (α4 subunit of α4β1 integrin) in recruitment of HSCs to injured tissue, HSC homing and HSC engraftment have been shown. Using multi-parameter flow cytometry, the results indicated that patients with prolonged engraftments had significantly lower α4 integrin on HSCs. Regression analysis showed an inverse association between α4 integrin and the probability of slow engraftment (18).

Studies on transplantation models blocking CD49d suggested that surface levels of VLA4 on HSCs influenced stem cell engraftment. This group previously showed that co-culture of HSCs with MSCs led to *ex vivo* expansion and engraftment enhancement of HSCs (19). Our data showed that co-culture of HSCs with MSCs plus FLT3-L led to more expression of CD49d compared to cocktails of cytokines. In another study, co-culture of HSCs with MSCs increased CD49d/VCAM1 mediating migration of primitive HSCs (20). Monitoring of FLT3-L signal showed that this molecule caused over expression of VLA-4 and VLA-5 on the HSC surface and consequently more adhesion of HSCs to MSCs which expressed VCAM-1 and ICAM-1 (7). Following this adhesion and signal transduction, HSCs mostly expanded without any differentiation. However, research has shown that soluble factors, adhesion molecules and matrix proteins supplied by MSCs are necessary for efficient division and maintenance of long-term stem cell population (21). Here efficacy of FLT3-L in the presence of MSCs as a feeder showed more CD34^+^ and LTC-IC fold increase than the routine combination of cytokines that included FLT3-L, TPO and SCF.

In the present study, the data demonstrated that harvested CD34^+^/CD38- cells significantly increased when HSCs were co-cultured on MSCs with FLT3-L and combinations of FLT3-L, TPO and SCF, compared to the cocktail of cytokine or feeder alone. Improvement of LTC-IC and HSC expansion overall in the group treated with a consistent combination of feeder and soluble factors showed *ex vivo* communication between MSCs and HSCs; consequently, stromal factors were released by MSCs, they simulated a hematopoietic niche and conserved HSC activity (5).

The MSCs are rare cells in the bone marrow that have crucial roles in HSCs development through secreting different cytokines and growth factors that include FL3, IL-6, TPO, GM-CSF and SCF. Chemokines expressed by MSCs such as CXCL12 and VCAM1 support long-term growth of HSCs without differentiation. According to the previous studies, co culture of HSCs with MSCs saves HSC’s pluripotency, because they provide survival signals for HSCs via adhesive molecules and cytokine signal regulation.

In addition to the role of mentioned cytokines and growth factors in HSC proliferation and survival, other molecules such as 5-aza-deoxycytidine (aza-D) and trichostatin A (TSA) also play play important roles in proliferation and survival of HSCs (22). A previous study has shown that co-expression of human FLT3-L and IL-6 by murine stromal cells supports human hematopoiesis without exogenous growth factors. IL-6 is potent activator of Signal transducer and activator of transcription 3 (STAT-3) causing altered expression of adhesion molecules (23). Notably, FLT3-L caused cell division of HSCs instead of quiescence, resulted expansion and survival improvement (24) and also is responsible for modulation of HSCs in steady-state adult hematopoiesis in mice (25). Furthermore, addition of cytokines, particularly FLT3-L, increased LTC-IC and proliferative capacity of HSCs in a synergistic manner. Binding of FLT3-L to the FLT3 receptor led to elevated HSC proliferation through both direct and indirect ways (26). The combination of FLT3-L with other cytokines noticeably increased TNC, CFU and LTC-IC compared with cytokines used alone. However FLT3-L in combination with MSCs as a feeder had the same effect as cytokine cocktails, particularly in expansion of LTC-IC which indicated that MSCs supplied the required cytokine. Previous study showed elevated *HoxB4* expression in hematopoietic stem and progenitor cells during expansion (27). Meaningful increase in *HoxB4* mRNA expression, confirmed the expansion of HSC in co-culture conditions in the presence of SCF, TPO and FLT3-L. Additional investigations on *HoxB4* activity in different stages of HSCs are suggested.

We propose that MSCs sufficiently supply the required molecules, cytokines and HSCs growth factors. On the other hand, FLT3-L stimulates HSCs expansion via activation of c-Kit, STAT5a and STAT5b molecules that coincides with other releasing factors by MSCs in a synergistic manner (28).

## Conclusion

This study showed that co-culture of FLT3-L clearly induced HSCs and LTC-IC expansion more than feeder-free culture with SCF, TPO and FLT3-L.
